# Rapidly Progressive Giant Malignant Phyllodes Tumor of the Breast: A Surgical Challenge

**DOI:** 10.7759/cureus.106935

**Published:** 2026-04-13

**Authors:** Georgia Xanthopoulou, Kyriaki Theodorolea, Christos Barkolias, Ilektra Spyrou, Nikolaos P Tasis

**Affiliations:** 1 Department of Surgery, Naval and Veterans Hospital of Athens, Athens, GRC

**Keywords:** breast cystosarcoma, giant phyllodes tumour, malignant phyllodes recurrence, malignant phyllodes tumour, phyllodes, phyllodes tumour

## Abstract

Malignant phyllodes tumors are rare fibroepithelial breast neoplasms that may pose significant diagnostic and surgical challenges, particularly when they reach extreme dimensions. Complete surgical excision with adequate margins remains the cornerstone of treatment. We report the case of a 65-year-old female who presented with a progressively enlarging mass of the left breast over an 18-month period. Clinical examination revealed a massive palpable lesion occupying nearly the entire breast, resulting in marked distortion of the breast architecture. Due to the extensive size of the tumor, conventional imaging modalities could not be adequately performed. Core needle biopsy demonstrated findings consistent with a malignant phyllodes tumor. The patient subsequently underwent left mastectomy, and histopathological examination confirmed complete excision of a 29 × 26 × 17 cm mass with negative, adequate margins. Eight months postoperatively, a local recurrence was identified along the mastectomy scar. The patient underwent wide local excision of the recurrent lesion followed by flap reconstruction. At three-year follow-up, the patient remains free of disease recurrence.

## Introduction

Phyllodes tumors represent less than 1% of all breast tumors and approximately 2%-3% of all fibroepithelial breast lesions. Based on their histopathological criteria, they are classified as benign, borderline, or malignant [1,2]. Malignant phyllodes tumors account for approximately 10%-30% of phyllodes tumors and demonstrate aggressive behavior, with a higher risk of local recurrence and, in rare cases, distant metastasis [3-5]. Preoperative diagnosis can be challenging because phyllodes tumors may mimic fibroadenomas both clinically and radiologically. Clinical features pointing toward phyllodes tumor are occurrence at an older age and a rapidly enlarging, painless mass without local lymph node involvement [6]. In cases where tumors grow rapidly or reach extreme dimensions, standard imaging modalities such as mammography or MRI may be technically difficult or impossible to perform [3].

Giant phyllodes tumors, usually defined as tumors larger than 10 cm in diameter, represent a rare clinical entity. Due to their large size and rapid growth, they frequently require mastectomy to achieve adequate surgical margins and complete resection [7]. We present a case of a giant malignant phyllodes tumor of the breast in a 65-year-old female, highlighting the diagnostic limitations associated with extreme tumor size, the surgical management strategy, and the surgical outcomes.

## Case presentation

A 65-year-old female presented with a progressively enlarging mass in the left breast that had been developing for over 18 months. Her past medical history included arterial hypertension under medication, three previous cesarean sections, and active smoking. She reported no known drug allergies, no previous breast cancer history, no hormone replacement therapy, and a negative family history. The patient denied pain, bleeding, nipple discharge, systemic symptoms, or weight loss.

Clinical examination revealed a giant palpable mass occupying nearly the entire left breast, causing significant distortion of the breast contour and pressure phenomena on the overlying skin. The skin was stretched but showed no ulceration, necrosis, erythema, or inflammatory changes. No palpable axillary lymphadenopathy was detected.

**Figure 1:**
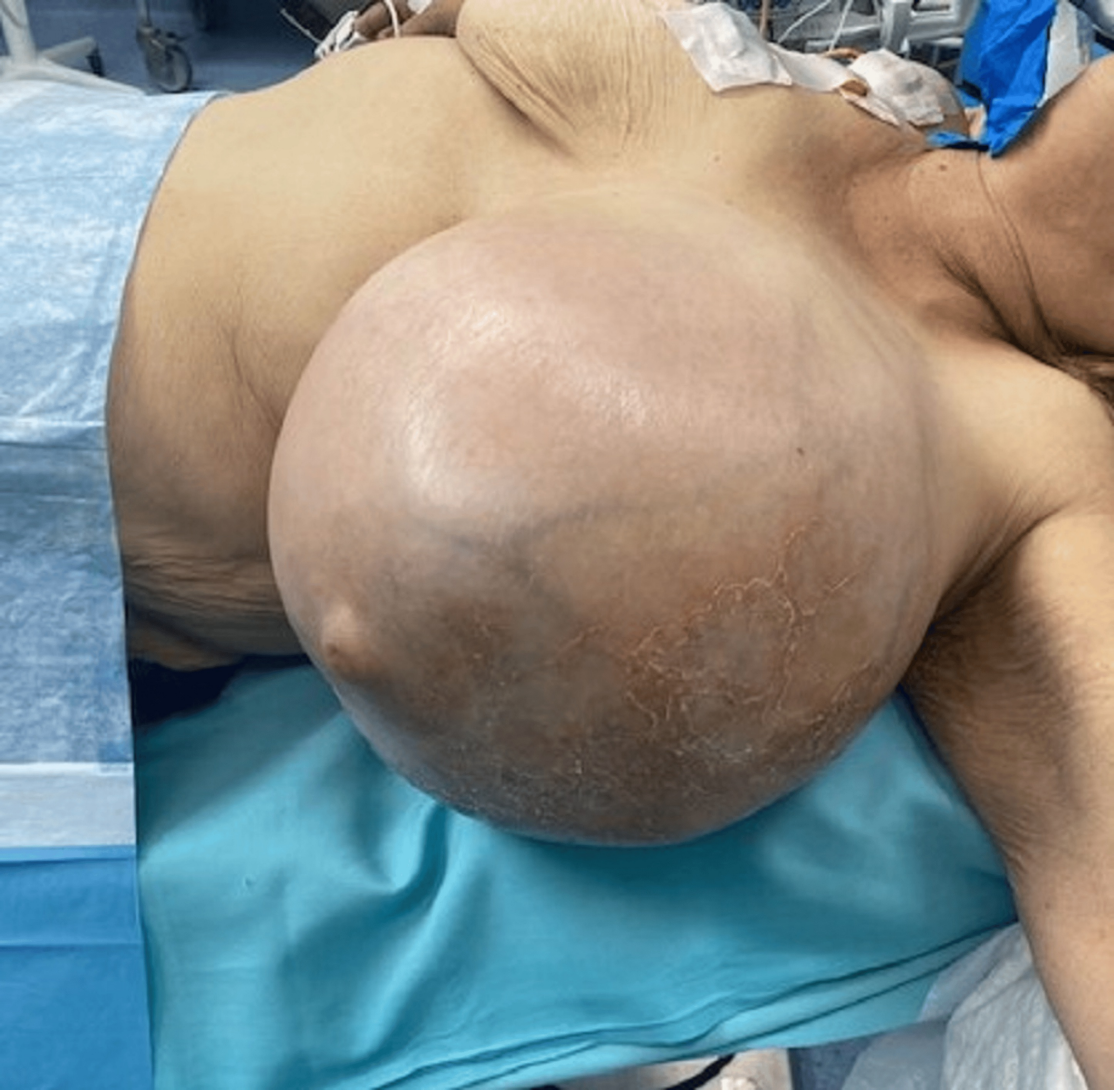
Giant phyllodes tumor

Conventional breast imaging proved difficult due to the massive tumor size. Mammography and magnetic resonance imaging could not be performed, as the breast could not be adequately positioned for imaging. Ultrasonography demonstrated a large, solid, heterogeneous mass occupying most of the breast, but the examination was not diagnostically conclusive due to the lesion’s size and heterogeneous internal architecture. The axillary ultrasound showed no suspicious lymph nodes (Figure 1).

Core needle biopsy demonstrated a malignant mesenchymal neoplasm composed predominantly of spindle-shaped stromal cells with marked nuclear atypia and high mitotic activity (>10 mitoses per 10 high-power fields). Areas of tumor necrosis were also observed. Immunohistochemical analysis showed the lesion to be Vimentin positive and CD34, MDM2, CD99, ER, PR, CD117, and β-catenin negative. The Ki-67 proliferation index was approximately 60%. These findings were consistent with a malignant mesenchymal tumor compatible with malignant phyllodes tumor with stromal overgrowth.

**Figure 2:**
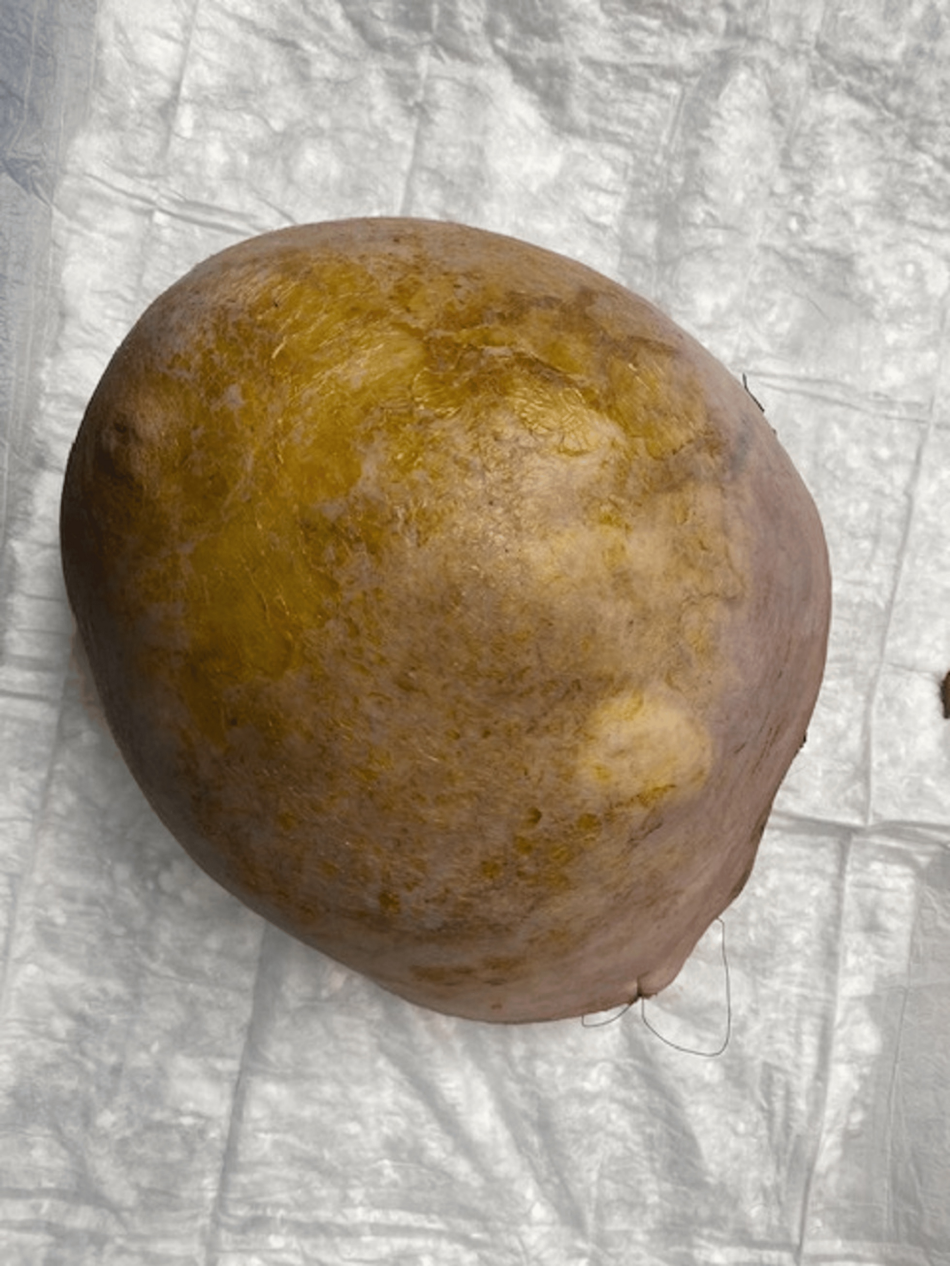
Surgical specimen of giant breast phyllodes tumor

Due to the tumor characteristics, its malignant nature, and its giant size, the patient was taken to the operating theater, where she underwent left mastectomy. No axillary procedure was performed due to the absence of clinically suspicious lymph nodes and the low incidence of lymphatic spread in phyllodes tumors (Figure 2). 

**Figure 3:**
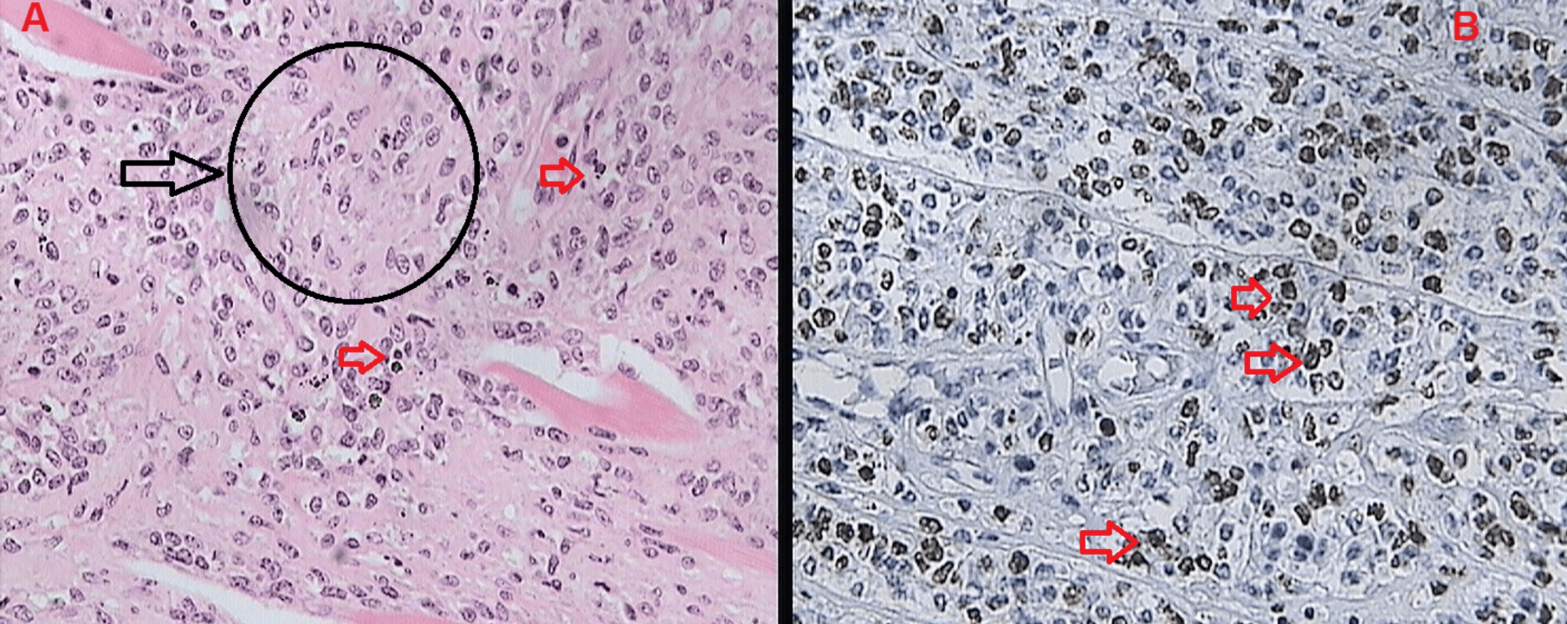
Malignant phyllodes tumor histology (A) Hematoxylin and eosin (H&E) stain (×40) showing hypercellular stromal overgrowth with marked nuclear atypia (black arrow and sample marked region) and increased mitotic activity (red arrows). (B) Ki-67 immunohistochemical stain (×40) demonstrating a high proliferative index (red arrows) in stromal tumor cells.

The final histopathology, macroscopically, revealed a giant tumor measuring 29 × 26 × 17 cm, weighing 2.95 kg, occupying nearly the entire breast. Microscopically, the lesion demonstrated a predominant sarcomatous stromal component with marked stromal cellularity and atypia, high mitotic activity, and stromal overgrowth, with Ki-67 over 60% (Figure 3). No epithelial component was identified in the examined sections. The tumor was completely excised with negative margins, with the closest margin measuring approximately 1.1 cm. The skin and nipple were not infiltrated. All other features were compatible with the preoperative biopsy demonstrating a malignant phyllodes tumor. 

**Figure 4:**
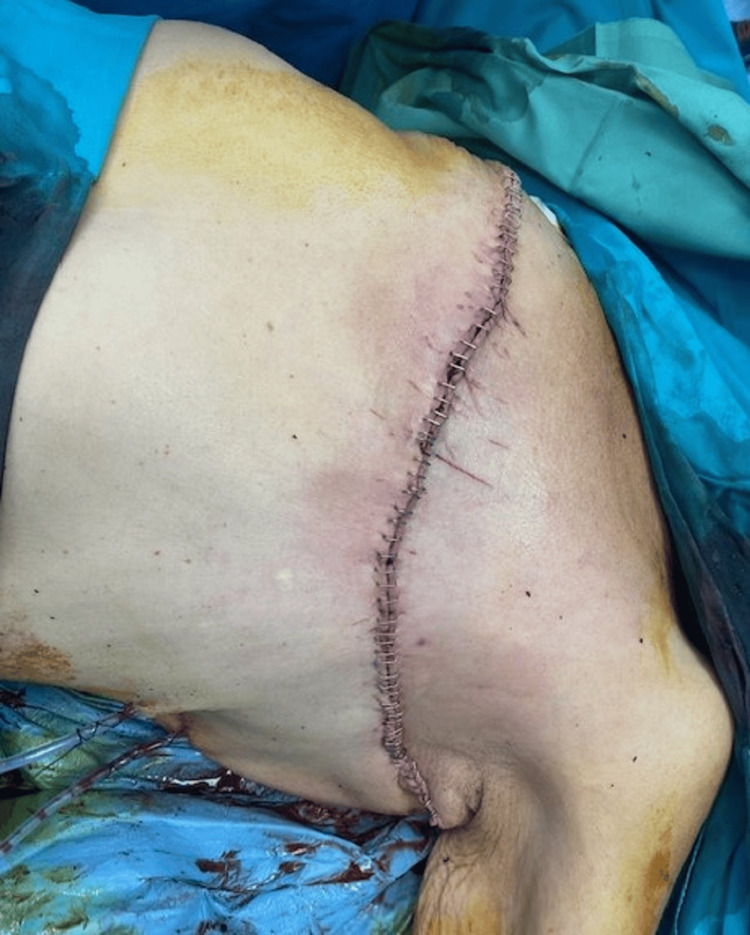
Immidiate post operative result

The postoperative course was uncomplicated. Adjuvant radiotherapy was recommended due to the tumor’s histological features; however, the patient declined radiation therapy (Figure 4).

Approximately eight months after the initial surgery, a 6 × 4 cm nodule developed along the mastectomy scar, consistent with local recurrence. The patient underwent wide local excision of the recurrent lesion, followed by reconstructive rectus abdominis myocutaneous flap surgery to restore chest wall contour. Histopathological examination confirmed recurrent malignant phyllodes tumor measuring 8.5 × 8 × 4 cm, which infiltrated the major thoracic muscle toward the axillary region and was histologically identical to the primary lesion. There was high mitotic activity (>10 mitoses per 10 high-power fields), along with multiple areas of tumor necrosis and a Ki-67 of 75%. All margins measured more than 2 cm. The second postoperative course was also uncomplicated.

The patient has been followed for three years following the second surgery, with ultrasound examination of the mastectomy site and both ultrasound and mammography of the contralateral breast yearly, and computed tomography of the chest, abdomen, and pelvis every six months. During this period, no further recurrence or distant metastasis has been detected.

## Discussion

Phyllodes tumors are uncommon breast neoplasms with heterogeneous biological behavior. They are characterized by a biphasic proliferation of epithelial and stromal components. Based on histopathological criteria, including stromal cellularity, nuclear atypia, mitotic activity, stromal overgrowth, and tumor margins, they are classified as benign, borderline, or malignant [1]. Their clinical course varies significantly depending on histological grade, tumor size, and adequacy of surgical excision. Malignant phyllodes tumors are characterized by marked stromal cellularity, nuclear pleomorphism, increased mitotic activity, and stromal overgrowth, reflecting their sarcomatous nature [2].

The diagnosis of phyllodes tumors relies on a combination of clinical examination, imaging studies, and histopathological evaluation. Radiologically, these tumors may resemble fibroadenomas on mammography and ultrasonography, which makes preoperative differentiation challenging. Core needle biopsy is therefore considered the most reliable method for establishing the diagnosis and is preferred over fine needle aspiration, but for inconclusive results, excision biopsy should be considered [6]. In cases where tumors reach extreme dimensions, as in our case, conventional imaging modalities such as mammography and magnetic resonance imaging may be technically difficult or impossible to perform due to limitations in breast positioning, field-of-view constraints, and inadequate compression or coil coverage [8,9]. Consequently, diagnosis in such cases often relies primarily on clinical assessment and tissue biopsy.

Surgical excision with negative margins remains the cornerstone of treatment. Current surgical guidelines recommend complete tumor removal with margins of approximately 1 cm whenever feasible, although the optimal margin width continues to be debated. Some recent studies suggest that narrower negative margins may still provide adequate local control, while others support wider excision in high-grade lesions [10,11]. In cases of giant phyllodes tumors, breast-conserving surgery is often not feasible due to the tumor’s size and the need to obtain adequate margins. Consequently, mastectomy is frequently required to achieve complete resection and minimize recurrence risk [7]. In our case, due to the extreme tumor size, a mastectomy was performed, and we achieved adequate 1 cm surgical margins. Unlike invasive breast carcinoma, axillary lymph node metastasis is extremely rare in phyllodes tumors because these tumors primarily spread through the hematogenous route. Therefore, routine axillary lymph node dissection is not recommended unless clinically suspicious nodes are present [3].

The role of adjuvant radiotherapy remains controversial. Several recent studies suggest that postoperative radiotherapy may reduce local recurrence rates in high-risk tumors. However, current evidence does not consistently demonstrate an improvement in overall survival [1,12]. Radiotherapy may be administered to high-risk MPT, such as tumors with high-grade histology (>30 mitoses per 10 high-power fields), size >5 cm, positive surgical margins, or recurrence [12]. While the epithelial component of most phyllodes tumors contains estrogen/progesterone receptor positivity, adjuvant endocrine therapy has no proven role in treatment [13].

Local recurrence remains one of the most important clinical challenges in the management of these tumors. Reported recurrence rates vary widely, with studies demonstrating recurrence in 7% to 30% of malignant phyllodes tumors [14]. Several factors have been associated with increased recurrence risk, including large tumor size, positive surgical margins, high mitotic index, and stromal overgrowth [13].

Surveillance in these patients is important and includes physical examination every six months for the first three years, then annually in years four and five, ultrasound every six months for three years, and annual mammography. Chest imaging, either chest X-ray or low-dose computed tomography (CT), is recommended every six months for two years and annually for years four and five, reflecting the 20% risk of distant metastasis, predominantly to the lungs [15].

In the present case, the patient underwent mastectomy with negative and adequate margins but declined adjuvant radiotherapy, even though the tumor presented high-risk characteristics. Local recurrence occurred eight months later along the mastectomy scar, which is consistent with previously reported recurrence intervals. Following surgical excision of the recurrence and reconstructive flap surgery, the patient again declined adjuvant radiotherapy, but she remained disease-free for three years, emphasizing the importance of long-term surveillance in these patients.

## Conclusions

Giant malignant phyllodes tumors are rare breast neoplasms that may present significant diagnostic and surgical challenges. When imaging modalities are limited due to tumor size, histological confirmation via core biopsy becomes essential for diagnosis. Complete surgical excision with adequate margins remains the cornerstone of treatment. Long-term close follow-up is mandatory due to the high potential for local recurrence. Early recognition and appropriate surgical management are critical for achieving favorable outcomes.
